# Quackery in Pakistan: a narrative literature review of a persistent public health threat

**DOI:** 10.1097/MS9.0000000000005360

**Published:** 2026-08-03

**Authors:** Waseem Sajjad, Sonali Priya Sahu, Sunaina Khan, Aswinipriya Bhaskar, Oisharja Fardeen Oishe, Zara Asif Dewan, Kabilesh Jothilingam, Najwa Nadeem, Shayan Nawaz, Seba Sayed Muhammed, Ammara Akram, Mubashra Aslam, Blessing DeNwigwe-Aggrey, Timothy Ojodare, Ihsan Qamar

**Affiliations:** a Department of Medicine, King Edward Medical University, Mayo Hospital, Lahore, Pakistan; b Department of Medicine, The Oxford Medical College, Hospital & Research Centre, Bangalore, India; c Department Medical Technology, Shifa Tameer-e-Milat University, Islamabad, Pakistan; d Department of Medicine, Georgian National University SEU, Tbilisi, Georgia; e Department of Medicine, Sylhet MAG Osmani Medical College & Hospital, Sylhet, Bangladesh; f Department of Medicine, Rashid Latif Medical College, Lahore, Pakistan; g Department of Medicine, BAU International University, Batumi, Georgia; h Department of Medicine, Faisalabad Medical University, Faisalabad, Pakistan; i Department of Medicine, University College of Medicine and Dentistry, Lahore, Pakistan; j Department of Medicine, Poonch Medical College, Rawalakot, Pakistan; k Department of Medicine, CMH Multan Institute of Medical Sciences, Multan, Pakistan; l School of Medicine, All Saints University School of Medicine, Roseau, Dominica; m Department of Medicine, University of Arizona, Tucson, Arizona, USA; n Department of Medicine, Spinghar Medical University, Kabul, Afghanistan

**Keywords:** malpractice, Pakistan, public health, quackery

## Abstract

**Introduction and background:**

Quackery remains a significant yet under-recognized public health threat in Pakistan. The term “quack” varies in definition across regions but commonly refers to individuals lacking formal training, legal registration, and authorization to provide healthcare services. As per Section 2(XXIX) of the Sindh Health Care Commission (SHCC) Act, 2013, a quack is defined as a pretender delivering healthcare services without registration with the Pakistan Medical Commission (PMC), National Council for Tibb (NCT), National Council for Homeopathy (NCH), or Pakistan Nursing Council (PNC).

**Objectives:**

This narrative review aims to explore the historical background, current prevalence, and contributing factors sustaining the practice of quackery in Pakistan, as well as to propose potential strategies for its control and eradication.

**Materials and methods:**

A narrative literature search was conducted across various databases, including PubMed, Science Direct, Embase, Google Scholar, and Scopus, along with official government documents from the Pakistan Medical and Dental Commission (PMDC), Provincial Health Commissions of Pakistan, the World Health Organization (WHO), and news outlets.

**Findings:**

Quackery continues to flourish in both rural and urban areas, largely due to systemic challenges such as poverty, illiteracy, lack of access to qualified medical services, and public mistrust of formal healthcare systems. While some quacks may provide short-term symptom relief, the long-term consequences often result in severe health complications or fatalities.

**Conclusion:**

Addressing the widespread menace of quackery in Pakistan requires a multifaceted and coordinated strategy. This includes stricter policy enforcement, expanded health education programs, strengthened primary healthcare infrastructure, and strategic media engagement for public awareness.

## Introduction

Although different regions like Europe, Asia, North America, South America, and Oceania have different definitions for “quack” and “quackery,” a common outlook for a quack is someone who lacks standard training, legal registration, and authorization for any domain of healthcare practice^[^1^]^. The word “quackery” originates from the 17th century, derived from “quack” (short for quacksalver), which itself comes from the Dutch kwakzalver, meaning “hawker of salve” or someone boasting about cures. It came to mean fraudulent or boastful medical practices^[^2^]^. According to Section 2(XXIX) of the Sindh Health Care Commission (SHCC) Act, 2013, a “quack” means a pretender providing healthcare services without having registration with the Pakistan Medical Commission (PMC) [formerly known as the Pakistan Medical and Dental Council (PMDC)], National Council for Tibb (NCT), National Council for Homeopathy (NCH), or the Pakistan Nursing Council (PNC)^[^3^]^. In this review, any unregistered/unlicensed providers delivering clinical care outside the legal scope are termed as quacks, and the practice is called quackery.

HIGHLIGHTS

- Quackery poses a significant but often overlooked public health risk in Pakistan due to the unauthorized practice of medicine by unqualified individuals.
- According to the Sindh Health Care Commission (SHCC) Act, 2013, a quack is someone who provides healthcare services without registration from recognized medical councils.
- The practice is widespread across both rural and urban areas, fueled by poverty, illiteracy, lack of access to qualified care, and distrust in the formal health system.
- Despite offering short-term relief, treatments by quacks frequently lead to severe complications or fatalities.
- Combating quackery requires a comprehensive strategy involving stricter regulations, public education, improved healthcare access, and media engagement.

In 1910, the Flexner Report was released in the United States, which stated that many medical schools were teaching incorrect science and medicine. The probe led to the closure of several poor-quality institutions^[^4^]^. In 1918, when the deadly influenza pandemic struck the world, people were terrified and desperate. Many individuals used poor, fake remedies like Eucapine ointment and Foley’s Honey and Tar to find relief because they did not know much about the sickness. However, at the same time, a major change in medicine was starting to happen. The disproportionate number of quality doctors compared to a large population was a leading cause for the public to seek quacks. These quacks then claimed to be experienced, using pseudo-scientific terms to deceive people^[^5^]^.

Globally, quackery takes numerous forms, including postmenopausal women claiming healing abilities in Europe, faith healers in Asia, deceptive “miracle” physicians in America, psychics in Africa, and non-scientific therapy providers in Oceania^[^6^]^. A 2016 survey found that in India, particularly in rural regions, untrained practitioners provide up to 70% of medical consultations. Despite government restrictions, quackery persists due to poor knowledge and inadequate enforcement^[^7^]^.

Pakistan is also encountering formidable obstacles in tackling quackery. Studies show that provinces like Punjab, Sindh, Khyber Pakhtunkhwa, and Balochistan are severely affected. In Punjab, an extensive suppression by the Punjab Healthcare Commission (PHC) closed around 69 266 quackery outlets from the inception of its services till April 2026^[^8^]^. A government survey in Sindh showed that more than 70% of healthcare consultants were unqualified, with around 3000 quacks working in different districts^[^3^]^. In Khyber Pakhtunkhwa, due to a widespread scarcity of trained practitioners, the people’s demand for quacks keeps increasing^[^9^]^. In Balochistan, the prevalence of quackery still exists despite anti-quackery laws by the provincial health commission, due to its large geographical area and limited resources^[^10^]^.

**Figure 1.**
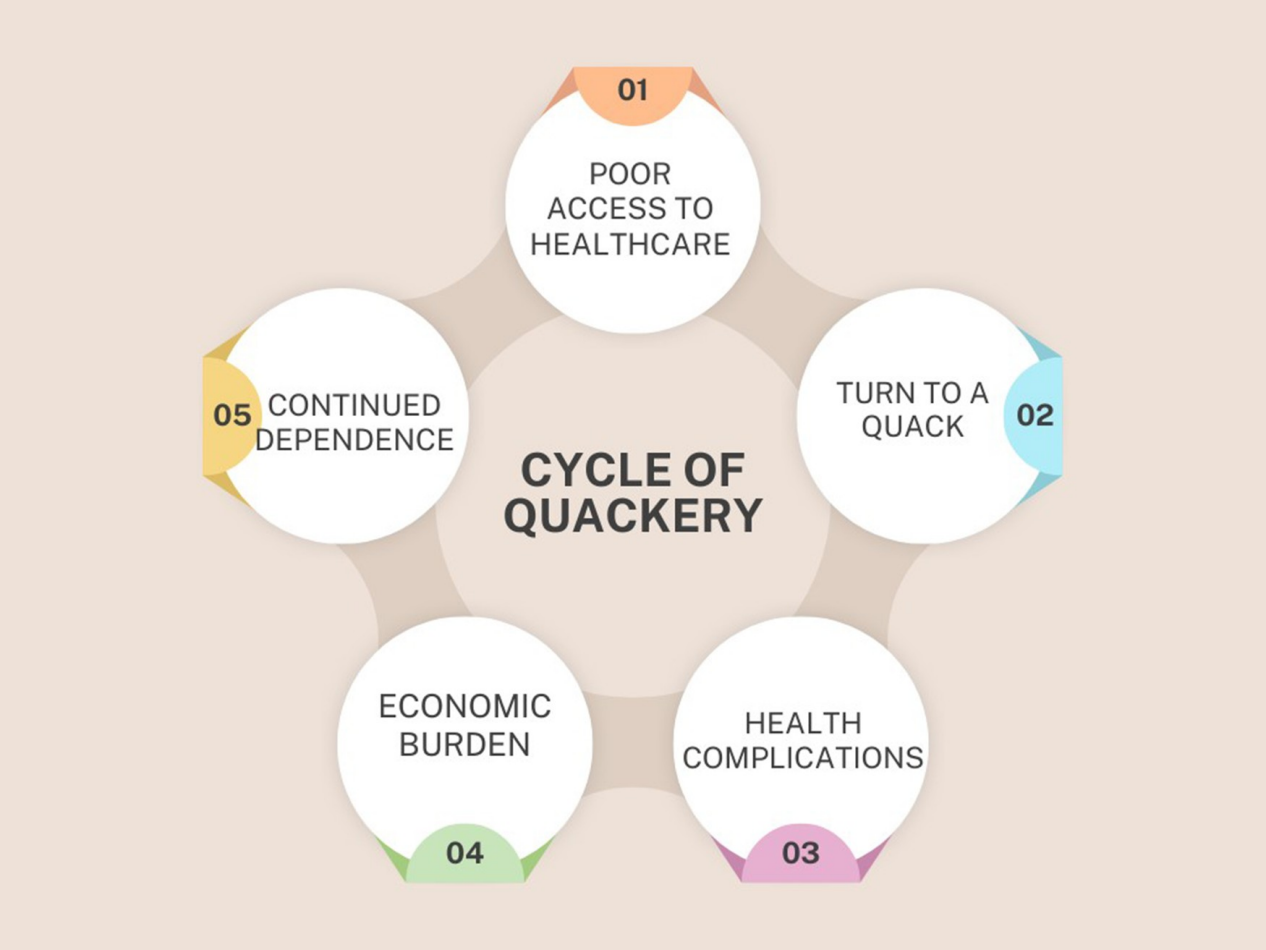
Cycle of quackery in developing countries^[^11^]^.

The continuation of quackery is caused by a number of interrelated factors, such as cultural beliefs, a lack of competent medical professionals, poverty, illiteracy, ignorance, weak law enforcement, and poor communication. Developing countries are more susceptible to quack practices because of these factors, as shown in Figure 1^[^11^]^.

Quackery is a major public health concern in Pakistan, as it causes the rapid spread of communicable diseases like human immunodeficiency virus (HIV) and hepatitis in urban and rural areas of Pakistan, and thousands of people lose their valuable lives due to wrong, non-scientific treatments^[^12^]^. We are addressing all these issues of quackery in this review to improve health care services, raise awareness, eradicate unauthorized practices, and save lives. This narrative review delves into the multifaceted dimensions of quackery in Pakistan – exploring its historical roots, current prevalence, and the social, economic, and cultural factors that sustain its existence.

This work has been done according to TITAN Guidelines 2025^[^13^]^.

## Materials and methods

This review was conducted as a narrative review in accordance with the Scale for the Assessment of Narrative Review Articles (SANRA) to ensure methodological rigor and transparency. A comprehensive literature search was conducted across various databases, including PubMed, Science Direct, Embase, Google Scholar, Scopus, official government documents from the Pakistan Medical and Dental Commission (PMDC), Provincial Health Commissions from Pakistan, and the World Health Organization (WHO), supplemented by gray literature sources such as government reports, policy documents, and reputable media outlets. The search was conducted from database inception to September 2025, restricted to English-language publications. Key search strings included combinations of terms such as “quackery,” “quacks,” “unlicensed practitioners,” “fake doctors,” “health fraud,” “Pakistan,” “illegal medical practice,” “traditional healers,” and “healthcare regulation in Pakistan,” using Boolean operators, and search strings were generated accordingly, as shown in Table 1. Records were screened in a two-stage process (title/abstract followed by full-text review), with duplicates removed. A total of 213 records were identified, of which 188 were screened, and 40 were finally included based on relevance to the topic. The credibility of gray literature sources was appraised based on source authority (e.g., government or institutional origin), transparency, and confirmation from multiple resources. The resultant abstracts and full-text papers were carefully screened and validated through group discussion. The date of the last literature search was 20 May 2025. Although this study does not follow a formal scoping review framework such as PRISMA-ScR, elements of structured reporting were incorporated to enhance clarity and reproducibility.

**Table 1:** Data sources of this study.

Database/Source	Keywords/Search terms	Details/Purpose
PubMed, Google Scholar, Science Direct, Embase, Scopus	Quackery, Fake Doctors, Health Fraud, Unlicensed Practitioners, Traditional Healers, Pakistan	Searched for peer-reviewed literature, academic journals, and scientific articles discussing the prevalence and impact of quackery.
PMDC Reports, Dawn News	Illegal Medical Practice, Quack Doctors	Reviewed official regulatory reports and news coverage to understand the scale and government stance on illegal medical practices.
Provincial Health Commissions	Quack Clinics	Analyzed provincial reports, especially from Punjab and Sindh, for registered cases and actions taken against unauthorized clinics.
World Health Organization (WHO)	Healthcare Regulation in Pakistan	Explored international guidelines and assessments on health system governance and regulation in the Pakistani context.

### Inclusion and exclusion criteria

This narrative review included journal articles that focused on the issue of quackery both globally and within Pakistan. It also incorporated relevant government reports and reliable gray literature. Studies and publications from all years and of various study designs were considered eligible for inclusion to ensure a comprehensive understanding of the topic. However, certain sources were excluded, including articles written in languages other than English, opinion pieces, and web content lacking evidence-based backing. Additionally, studies that were unrelated to public health concerns or the regulation of healthcare practices were excluded from the review.

### Ethical considerations

This study relied solely on publicly available information and did not involve the collection of primary data or the use of any patient-specific information. Therefore, ethical committee approval was not required. All data and content referenced in the review were gathered from credible sources and have been appropriately cited to maintain academic integrity and transparency.

### Quality appraisal/bias acknowledgement

Although this review is narrative in nature and does not employ a formal risk-of-bias assessment, we acknowledge the variability in quality among the included sources. The literature reviewed comprises peer-reviewed articles, observational studies, policy documents, gray literature, and media reports, each differing in methodological rigor and reliability. Consequently, findings should be interpreted with caution, as some reports may reflect contextual or reporting bias. Nevertheless, by triangulating evidence from multiple data types and sources, this review aims to provide a balanced and comprehensive understanding of the issue while recognizing the inherent limitations of the available evidence base.

## Forms of quackery prevalent in Pakistan

### Unlicensed medical practitioners

In Sindh, Pakistan, quacks – unlicensed medical practitioners – are a significant problem, particularly in Hyderabad, Mirpur Khas, and Shaheed Benazir Abad. In addition to ignoring basic infection control and frequently reusing IV drip sets and syringes, many private health personnel lack proper qualifications, which raises the danger of patient infection. To legitimize these clinics, some certified MBBS physicians shockingly rent out their identities^[^14^]^. According to the Pakistan Health Parliament, a project of Oliver’s Trust, quackery is a significant issue in Pakistan, with up to 900 000 quacks engaging in unlawful practice as of May 2026^[^15^]^. A lack of doctors in remote areas, poverty, inadequate access to healthcare, lax law enforcement, and low public awareness are some of the main causes. Misdiagnosis, delayed treatment, illness transmission, antibiotic resistance, and mistrust are the results. By offering qualified, reasonably priced care, increasing awareness, and facilitating data collection, telemedicine provides an answer. Investments in telemedicine, more stringent legislation, and public education are necessary to combat quackery^[^15^]^.

### Fake traditional healers (pirs, hakeems, spiritual healers)

Due to illiteracy and historical customs, people living in slum areas turn to “aamils” for assistance with problems, illnesses, and quandaries. They firmly believe in the spiritual abilities of these so-called “aamils,” who, in return, give them “taweez” (amulets) in exchange for money and proclaim that they will cure all of their afflictions, including infertility, marital problems, business difficulties, domestic disputes, and retaliation^[^16^]^. Since January 2018, four distinct kidney stones from various places in Pakistan have been studied using FTIR spectroscopy. Chemical studies revealed no common components like calcium, oxalate, or magnesium, and their spectra did not match any of the 756 NICODOM reference spectra. Every patient had sought the advice of various spiritual healers, or “pirs,” who asserted that the stones were the result of black magic and could be extracted without the need for medication or surgery. In Pakistan, where “pirs” and “aamils” assert magical healing abilities, this demonstrates the widespread use of non-scientific spiritual practices^[^17^]^.

### Unauthorized dental and surgical services

Unlicensed practitioners of dental quackery employ inappropriate instruments and materials, such as self-cure acrylic, to perform risky procedures that result in financial, emotional, and physical injury. These practices lead to dental damage, incorrect diagnoses, and the spread of HIV/AIDS and hepatitis. Quacks frequently rely on dentists or internet resources to provide quick, painless solutions. Although it is less common in wealthy nations, dental fraud remains a global issue^[^18^]^.

### Sale of counterfeit medicines

This WHO alert refers to falsified DOW USP/EP PROPYLENE GLYCOL detected in Pakistan in August 2024 and notified to WHO in September 2024. Authentic DOW USP/EP PROPYLENE GLYCOL is a raw material (excipient) utilized in pharmaceutical and other manufacturing processes, adhering to the standards of the United States and European Pharmacopoeias (USP/EP) for medicinal use^[^19^]^. Although pharmaceuticals are regulated by health organizations such as the Drug Regulatory Authority of Pakistan (DRAP), counterfeit drugs continue to be a rising issue. Authorities in Karachi confiscated fake cefixime and aspirin powders that were repackaged as name-brand goods. A state-wide alert was also issued following the discovery of an illicit factory manufacturing necessary medications such as cephalosporins and meropenem^[^20^]^.

### Homeopathy and herbal quackery without evidence-based practice

**Figure 2.**
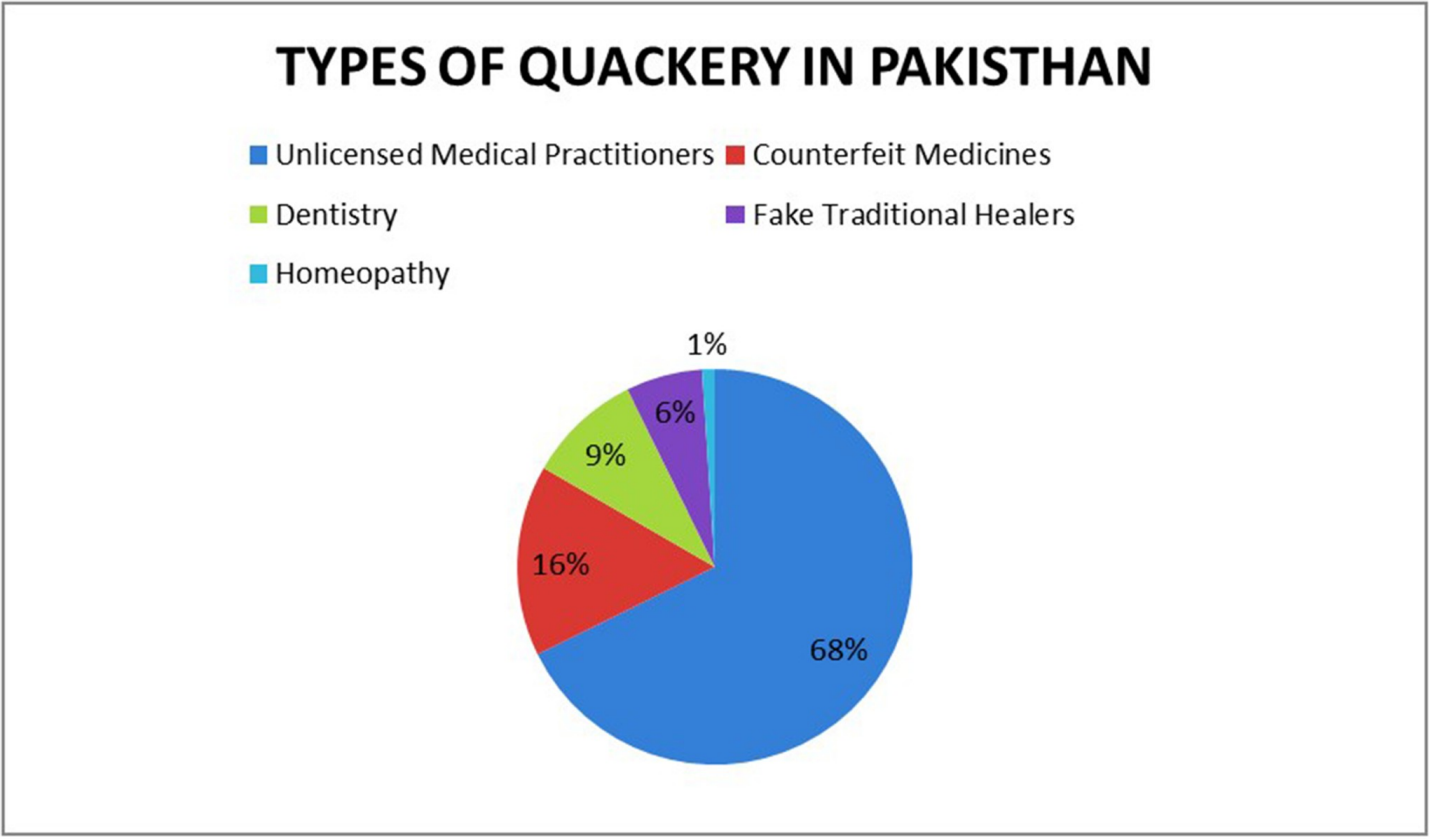
Overview of the prevalence of quackery in Pakistan^[^8^]^.

Since prescribing allopathic drugs is outside their legal scope of practice, homeopaths in Sindh, Pakistan, are practicing quackery. Financial benefits, patient demand for quicker results, and the prestige of allopathic treatment are some of the causes driving this illegal activity. In Pakistan, homeopathy is still widely practiced despite its worldwide decline, in part because many are unaware of its legal restrictions. Patients frequently trust these practitioners because they are affordable and unaware of the dangers of quackery, which makes anti-quackery initiatives difficult^[^21^]^ (Table 2 and Fig. 2^[^3,8^]^).

**Table 2:** Year-wise and region-wise data about types of quackeries.

Year	Region with highest quackery	Most common type of quackery	Total quack clinics sealed
2023–2024	Lahore	Unlicensed medical practitioners	7241
2022–2023	Lahore	Unlicensed medical practitioners	6505
2021–2022	Lahore	Unlicensed medical practitioners	5249
2020–2021	Shaheed Benazirabad	Unlicensed medical practitioners	6561
2019–2020	Lahore and Shaheed Benazirabad	Unlicensed medical practitioners	23 000

## Factors contributing to the rise in quackery

**Figure 3.**
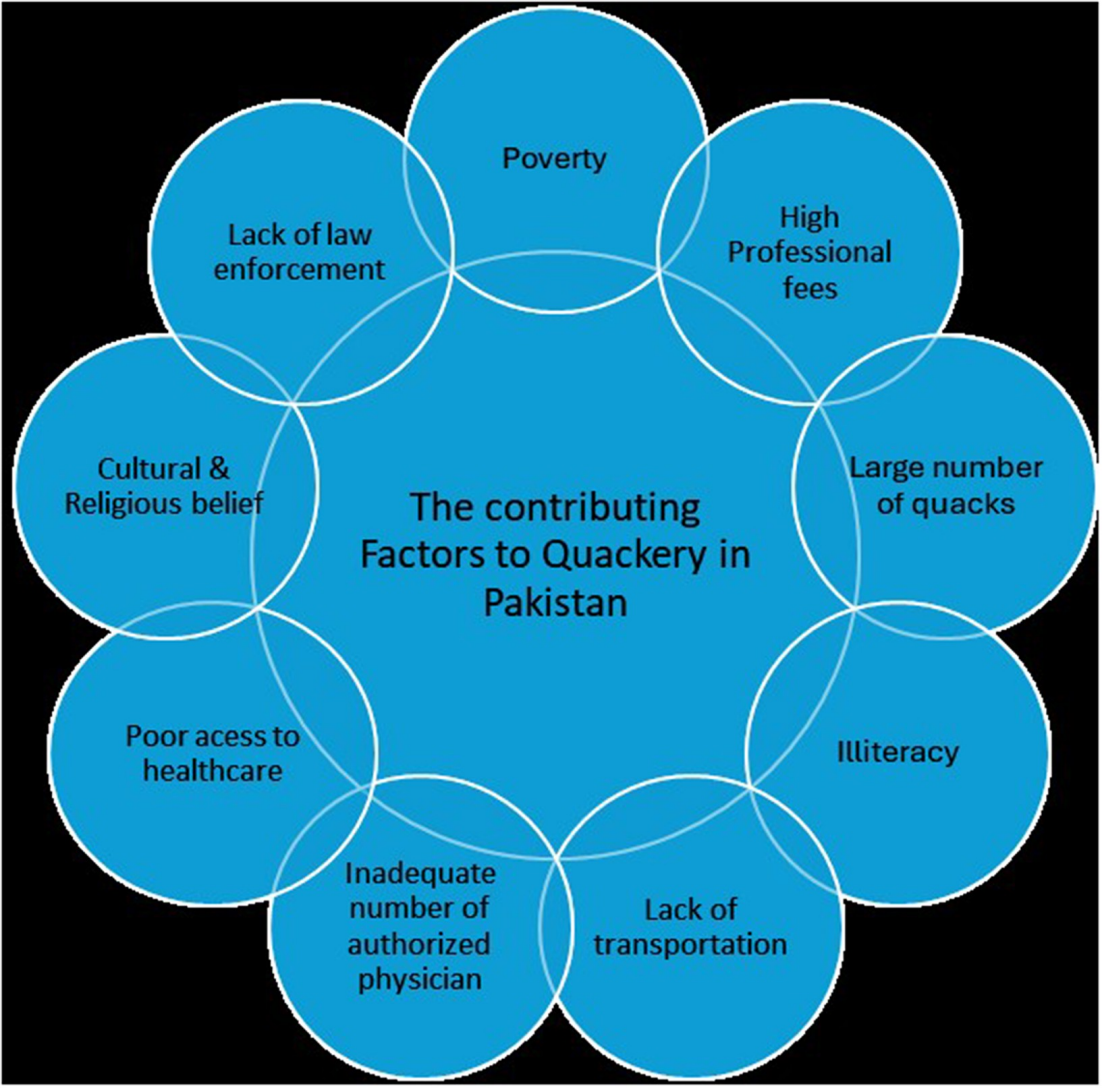
Factors contributing to the rise of quackery in Pakistan (according to a study conducted in D.I. Khan, Khyber Pakhtunkhwa)^[^22^]^.

In order to evaluate the factors that lead to quackery, a questionnaire-based survey was conducted in Dera Ismail Khan’s rural, urban, and semi-urban regions on unprofessional practices and the ratio of quacks to certified professionals. A sample size of 150 was used, and separate questionnaires were designed for quacks and the general public. The primary causes of quackery in rural areas were the large number of quacks (50%), poverty (60%), high professional fees (56%), illiteracy (42%), lack of transportation (40%), and poor access to healthcare (40%), as shown in Figure 3^[^22^]^. These factors were lower in urban and semi-urban areas. The quack-to-professional ratio, according to a study conducted in Dera Ismail Khan, KPK, was highest in rural areas (157:10), followed by semi-urban areas (29:15) and urban areas (50:35)^[^22^]^.

This disparity is strongly supported by the data collected from the Pakistan Medical and Dental Council, which states that an estimated 600 000 quacks are currently operating in Pakistan as of May 2025. Though this number varies from that of the Pakistan Health Parliament (which states it as 900 000), it is still high enough to be worth concerning^[^3,15^]^. Approximately one-third of them are based in the Sindh province, with nearly 40% of them practicing in Karachi. A key factor contributing to the widespread reliance on quacks is the pronounced disparity between the availability of licensed healthcare professionals and the needs of the population. Statistics from the Sindh Healthcare Commission indicate that one quack is available for every 350 individuals, whereas one licensed practitioner serves approximately 1290 people, as per SHCC data from May 2025^[^3^]^. This significant gap in accessibility, coupled with the comparatively higher cost of consulting licensed professionals, creates fertile ground for quackery, as noted by SHCC in May 2025^[^3^]^. This number may vary significantly across other provinces and at the national level, requiring robust and regular data management as well as unbiased periodic surveys. People with low income and a lack of proper knowledge and awareness often end up seeking assistance from quacks^[^5,23,24^]^.

Dietary patterns and women’s symptomatic burden may influence care-seeking preferences and vulnerability to unproven therapies^[^25^]^. Disruptions to essential services and supply chains following disasters can heighten reliance on informal or unregulated providers^[^26^]^. Marginalized groups with poor nutritional status and limited access to formal care are more likely to seek alternative providers^[^27^]^.

Failure of regulatory bodies to implement rules and laws, along with their lack of interest, serves as a key barrier to eradicating quacks. A qualitative research design was developed under the Punjab Healthcare Commission’s (PHC) anti-quackery mandate to identify these challenges. The research also revealed deficiencies in strategic and operational planning, delayed or absent top-level decision-making, along with a shortage of trained personnel within the Anti-Quackery Cell, undermining its effectiveness in the field^[^28^]^.

Quacks often include spiritual healing in healthcare practices in order to take advantage of cultural norms and religious beliefs. They use prayers, rituals, sorcery, and supernatural beliefs such as the evil eye, bad luck, and “Jinni” possession to attract individuals with limited education. Furthermore, traditional systems like homeopathy, Veda, and Hakeem are legally recognized forms of healthcare in countries such as Pakistan, Bangladesh, India, and Iran^[^1,29,30^]^. These systems often coexist with modern medical practices, leading to a blending of methodologies that can confuse patients regarding the best course of treatment. As a result, individuals may turn to these traditional practices despite the potential risks associated with unregulated and unproven treatments.

Table 3 illustrates the key quantitative and qualitative findings from the available literature cited above in this section on quackery in Pakistan, including prevalence estimates, regional disparities, contributing factors, and systemic challenges. The data are derived from survey-based studies, governmental and institutional reports, and published literature, as cited.

**Table 3:** Summary of Available Data on Quackery in Pakistan.

Indicator	Setting/Region	Key findings	Source
Prevalence factors contributing to quackery	Rural Dera Ismail Khan	High burden attributed to poverty (60%), high professional fees (56%), large number of quacks (50%), illiteracy (42%), lack of transportation (40%), and poor healthcare access (40%)	^[^22^]^
Quack-to-professional ratio	Rural Dera Ismail Khan	157:10 (highest disparity)	^[^22^]^
Quack-to-professional ratio	Semi-urban Dera Ismail Khan	29:15	^[^22^]^
Quack-to-professional ratio	Urban Dera Ismail Khan	50:35	^[^22^]^
Estimated number of quacks	National (Pakistan)	~600 000 reported by Pakistan Medical and Dental Council; ~900 000 estimated by Pakistan Health Parliament	^[^3,15^]^
Provincial distribution	Sindh	~33% of quacks located in Sindh; ~40% of these in Karachi	^[^3^]^
Population coverage	Sindh	One quack per ~350-person vs one licensed practitioner per ~1290 person	^[^3^]^
Socioeconomic determinants	National	Low income, poor awareness, and limited healthcare access increase reliance on quacks	^[^5,23,24^]^
Additional vulnerability factors	Various contexts	Dietary patterns, women’s symptom burden, disasters, and disrupted healthcare systems increase dependence on informal providers	^[^25–27^]^
Regulatory challenges	Punjab	Poor in enforcement, poor strategic planning, delayed decision-making, and shortage of trained personnel in Anti-Quackery Cell	^[^28^]^
Cultural and traditional influences	South Asia (including Pakistan)	Use of spiritual healing, belief in supernatural causes, and coexistence of traditional systems (homeopathy, Hakeem, Veda) with modern medicine	^[^1,29,30^]^

This table summarizes key quantitative and qualitative findings from available literature on quackery in Pakistan, including prevalence estimates, regional disparities, contributing factors, and systemic challenges. The data are derived from survey-based studies, governmental and institutional reports, and published literature, as cited.

## Public health impact of quackery

Quackery is a complex phenomenon with multifaceted consequences on healthcare systems, which include health, social, and economic impacts. Observed health consequences are increased mortality (avoidable death) and morbidity; decreased medical effectiveness; denied or delayed effective treatments; increased risk of poisoning, adverse reactions, or resistance to drugs; and disease mongering. This negatively impacts social trust, with the public losing trust in science and medicine, medical professionals, and the broader healthcare profession, which in turn fuels disease burden, making the community a breeding ground for preventable disease outbreaks as well. Furthermore, it contributes to the imminent failure of future public health interventions in that community. It also has negative economic effects on healthcare systems through financial deficits, inefficient resource utilization, inadequate health coverage, and increased financial burden. These multidimensional consequences are detrimental for a developing nation like Pakistan, as they not only diminish current health progress and economic prosperity but also risk future development^[^5^]^.

**Figure 4.**
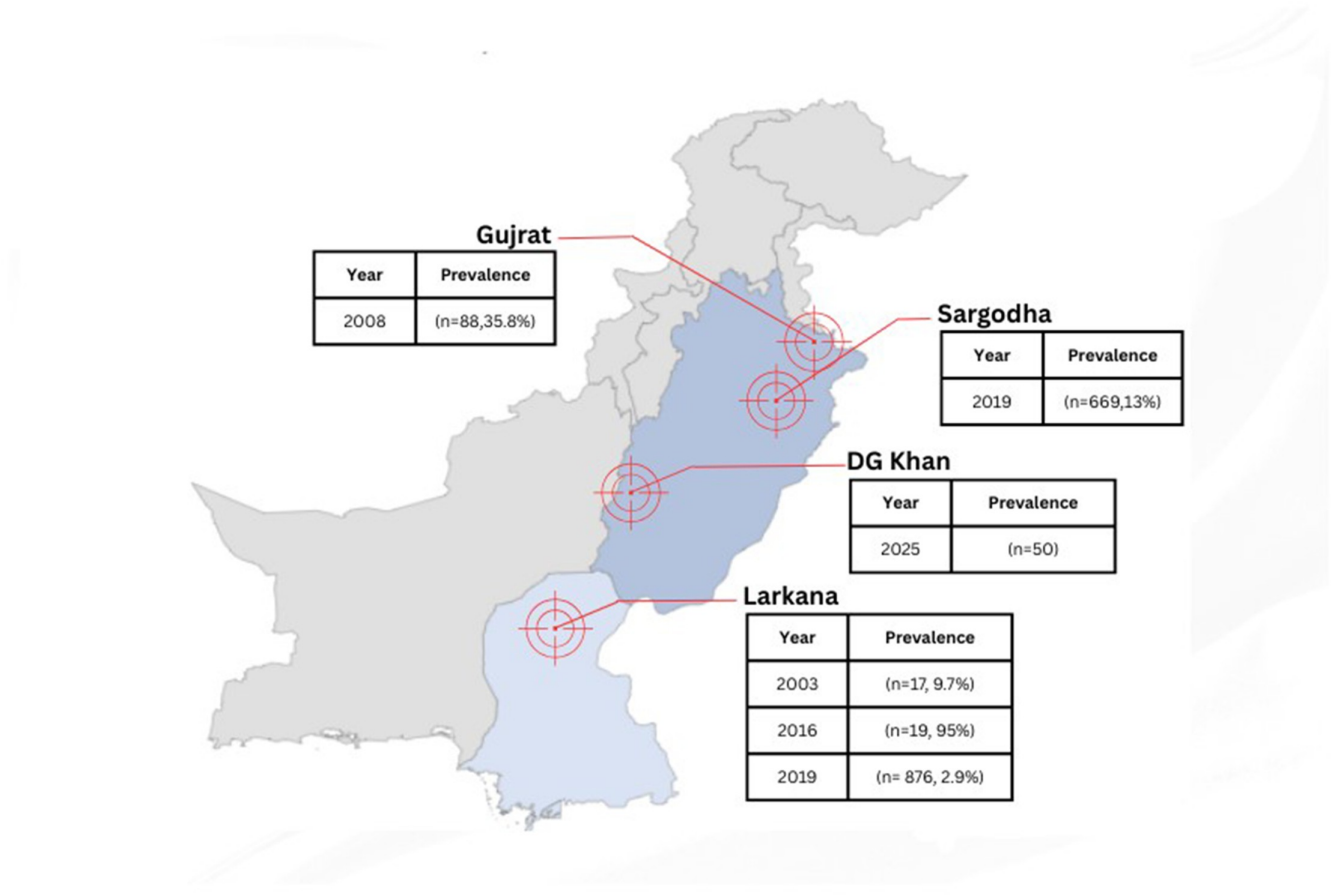
Timeline of HIV outbreaks in Pakistan to date^[^31^]^.

HIV outbreaks have been one of the major public health tragedies in Pakistan. Large-scale outbreaks have been reported in Gujrat, Sargodha, and Larkana (Fig. 4)^[^31^]^. The first outbreak in Sargodha was reported in 2018 with a prevalence of 1.3%, which rapidly spread and reached 13% in 2019. In the outbreak in the Larkana region, HIV infection was reported in 604 children and 135 adults. Most of the infected children did not have HIV-positive parents. The reuse of contaminated needles and malpractice by quacks in private settings were identified as the main contributing factors to these HIV outbreaks^[^31^]^. Moreover, it has been observed that patients requiring frequent blood transfusions, such as thalassemia patients in Pakistan, have a high prevalence of hepatitis B, hepatitis C, and HIV infections, which studies suggest were acquired through unsafe blood transfusions^[^32^]^.

Antimicrobial resistance (AMR) has emerged as one of the top global health threats of the 21st century, and the COVID-19 pandemic has only made it worse. The overuse and misuse of antibiotics globally during the pandemic turned the existing AMR threat into a catastrophic situation^[^33^]^. This has particularly affected low- and middle-income countries (LMICs) like Pakistan, where quackery is rampant. Since 2014, Pakistan has recognized antimicrobial resistance as a national concern. Pakistan observed a 65% increase in the consumption of antibiotics between 2000 and 2015. The consumption saw an alarming increase during the pandemic, which poses a significant threat to the local healthcare system and development. In Pakistan, one can easily gain access to various medications without a prescription. Therefore, it is easier for quacks to operate and for the public to self-medicate with antibiotics and steroids. Until now, some regulatory efforts have been made to control quackery and manage over-the-counter medication; however, the response has been concentrated on major cities and was prompted by grave public health events. Therefore, a comprehensive regulatory and legal approach covering the entire country is needed to restore the local community’s health and ensure future health development^[^34,35^]^.

Quackery ails Pakistan despite raids and retribution by the Health Commission in each province, mainly due to the lack of knowledge about the adverse effects among the masses. The cases reported are heartbreaking, leaving a substantial impact on the quality of life of the victims.

In July 2023, an article was published that recalled encountering a large number of children presenting with signs and symptoms similar to Cushing’s syndrome, including excessive weight gain, moon facies, and stunted growth in Pakistan. It was stipulated, upon investigation, that they had been treated by local quacks with steroids for various illnesses, such as viral cough, pneumonia, and skin infections^[^36^]^.

In April 2019, an HIV outbreak was reported in the town of Ratodero in the Larkana district, Sindh. Fifteen children with persistent fever were sent for HIV testing, which came back positive. Screening revealed 157 HIV-positive cases, including 30 adults and 127 children. An inquiry revealed the reuse of contaminated disposable syringes by someone impersonating a doctor^[^37^]^.

In March 2019, the anti-quackery department raided a clinic in the suburbs of Rawalpindi. It was run by a quack who had a qualification in homeopathy, yet he was administering veterinary medicines to humans with unsterilized equipment^[^38^]^

Sadly, some of these clinics are backed by registered medical practitioners who charge these quacks monthly “rent” to run the clinics under a guise. In 2018, the District Health Authority took up the case of three doctors from a Tehsil Headquarter (THQ) Hospital in Punjab to the Pakistan Medical and Dental Council in order to cancel their postgraduate training degrees. The doctors were accused of providing false affidavits in support of a quack running a clinical laboratory. Another quack was nabbed for performing surgeries without being qualified, which included Cesarian sections. Critical patients had to be shifted to the THQ Hospital, and the illegal operation theater was sealed^[^39^]^.

Similarly, there are dental clinics run by technicians, providing cheap solutions to dental ailments, including unhygienic tooth extractions and scaling. These practices lead to serious infections of the jaw and, ultimately, systemic effects.

In 2024, the Islamabad Healthcare Regulatory Authority (IHRA) sealed a hospital in the Tarnol area of Islamabad after expired injections, broken vials, expired blood sample tubes, and expired medicines were found. Additionally, there was no waste management system^[^40^]^.

As of 12 May 2025, the Punjab Health Commission has sealed 58,805 quackery outlets. According to the PHC Anti-Quackery Strategy 2015, there is one allopathic registered doctor available for about 1290 patients, whereas one unqualified practitioner is available for over 350 patients^[^8^]^. This shows the increasing amount of access these people have in their local communal area.

## Legal and regulatory frameworks in Pakistan:

### Existing laws against quackery

The Punjab Healthcare Commission (PHC) is empowered by the Punjab Healthcare Commission Act (2010) as outlined in Table 4 to oversee healthcare services, including disciplining unlicensed professionals. In order to eradicate unlicensed medical practices, it has also created an anti-quackery policy. No one may practice medicine or dentistry unless they are a registered medical or dental practitioner, as stated in Sections 28 A and 28 B. Additionally, it imposes a fine of up to 200 000 PKR, which cannot be less than 100 000 PKR, and a jail sentence of up to 2 years, but not less than 6 months^[^8^]^. In order to combat quackery, the Sindh Healthcare Commission (SHCC) was established under the Sindh Healthcare Commission Act of 2013. Its Anti-Quackery Directorate inspects establishments without the required licenses and has the authority to close them or impose fines. The Anti-Quackery team’s authority and duties are outlined in Section 39 of the 2017 SHCC regulations^[^3^]^. An Anti-Quackery Section was established as part of the relevant Directorate by the Khyber Pakhtunkhwa Health Care Commission (Anti-Quackery) Regulations, 2022, under the direction of the Director duly appointed by the Commission^[^10^]^. The Balochistan Healthcare Commission Act No. XII (2019) was put into effect to enhance the standard of healthcare services and outlaw quackery in all of its manifestations^[^12^]^.

**Table 4:** Summary of anti-quackery regulations in Pakistan by Province.

Province	Regulatory body	Key legal frameworks
Punjab	Punjab Healthcare Commission	PHC Act 2010; Anti-Quackery Regulations 2016 & 2017
Sindh	Sindh Healthcare Commission	SHCC Act 2013; Anti Quackery Rules 2017 (Section 39)
Khyber Pakhtunkhwa	KP Healthcare Commission	KPHCC Anti-Quackery Regulations 2022
Balochistan	Balochistan Healthcare Commission	BHC Act 2019 (Act No. XII of 2019)
Federal (Islamabad)	Pakistan Medical Commission	PMC Act 2020

### Role of regulatory bodies

The Pakistan Medical Commission (PMC) is responsible for regulating and registering medical professionals in the country. In order to take disciplinary action against unauthorized or unethical actions, it has enforcement regulations (2021)^[^41^]^. Every province has a healthcare commission (PHC, SHCC, KPHCC, or BHC) that is responsible for implementing anti-quackery policies, including licensing, public awareness campaigns, and inspections.

### Challenges in enforcement

Despite legal frameworks, quackery remains prevalent. For instance, in Sindh, numerous fake doctors operate clinics without fear of repercussions. A lot of unregistered private hospitals run in Hyderabad, while even registered ones fail to display approved fee charts as required by law^[^42^]^. Provincial commissions often face limitations in manpower and resources, which obstruct effective implementation and monitoring of quackery. Additionally, a lack of public knowledge about the dangers of quackery contributes to its persistence, as individuals continue to seek treatment from unqualified practitioners.

### Legal gaps in combating quackery in Pakistan

The Pakistan Journal of Medical and Health Sciences published research that addresses the various negative effects of quackery, as well as the difficulties the Punjab Healthcare Commission (PHC) has faced in carrying out its anti-quackery mandate. It highlights how important it is to change laws and bolster enforcement^[^28^]^. The inadequate regulatory frameworks for private general practitioners in Pakistan’s rural districts are brought to light via an exploratory qualitative study. It emphasizes how these regulatory inadequacies affect the provision of healthcare^[^43^]^. This study examines the causes of Sindh’s public preference for quacks, pointing out the lack of enforcement of laws prohibiting the sale of medications to unlicensed practitioners^[^12^]^. Several facets of quackery practices, including legal definitions and laws, are analyzed in a scoping review published in the Journal of the Pakistan Medical Association. It highlights the necessity of precise legal frameworks and uniform application of the law in various geographical areas^[^12^]^.

## Efforts made to curb quackery

### Crackdown campaigns by provincial healthcare commissions

As a result of the Punjab Healthcare Commission’s (PHC) vigorous anti-quackery approach, more than 44 600 treatment facilities have been inspected, and over 19 250 quackery outlets have been sealed, as shown in Figure 7. The province’s efforts to eradicate unlicensed medical practices include these measures^[^8^]^. The Sindh Healthcare Commission (SHCC) has stepped up its enforcement and inspection efforts, especially in districts like Khairpur, Ghotki, and Sukkur. Section 39(G&H) of the SHCC Regulations 2017 has resulted in the sealing of numerous quack clinics, demonstrating the commission’s determination to eradicate quackery^[^3^]^. To combat untrained practitioners, the Khyber Pakhtunkhwa Healthcare Commission (KPHCC) has created regulations and established an Anti-Quackery Cell. To guarantee that illegal establishments are dealt with, their approach entails licensing and registering skilled individuals, as well as putting in place a responsive complaints management system^[^9^]^. With the goal of enhancing healthcare quality and outlawing quackery throughout the province, the Balochistan Healthcare Commission (BHC) is governed under the Balochistan Healthcare Commission Act 2019.

### Public awareness programs

To inform the public about the risks associated with quackery, the PHC has arranged awareness campaigns and seminars. The purpose of these programs is to educate the public on the value of obtaining care from qualified medical professionals. The SHCC has held public awareness events with an emphasis on gathering precise quack statistics, identifying places where quacks are common, and working with law enforcement. The message is amplified through positive media support in addition to these initiatives^[^44^]^. In 2022, public seminars, awareness walks, and the use of various media platforms were utilized under the KPHCC’s anti-quackery laws to spread knowledge about the health issues caused by quackery and the measures taken to eradicate it^[^45^]^.

### Role of NGOs and media

Pakistani media sources have played a significant role in raising awareness of the dangers of quackery. Public awareness of the risks associated with receiving treatment from unqualified practitioners has increased as a result of investigative reports and news pieces that have exposed their operations. The Economist, for example, described Punjab’s Anti-Quackery Department’s attempts to identify and shut down unlicensed clinics, highlighting the media’s role in educating the public and assisting with regulatory actions^[^46^]^. Similarly, The Express Tribune covered the District Health Authority’s efforts to reduce the number of quacks practicing in public areas, emphasizing the importance of consulting licensed medical professionals and the role that the media plays in disseminating information about enforcement actions^[^47^]^.

**Figure 5.**
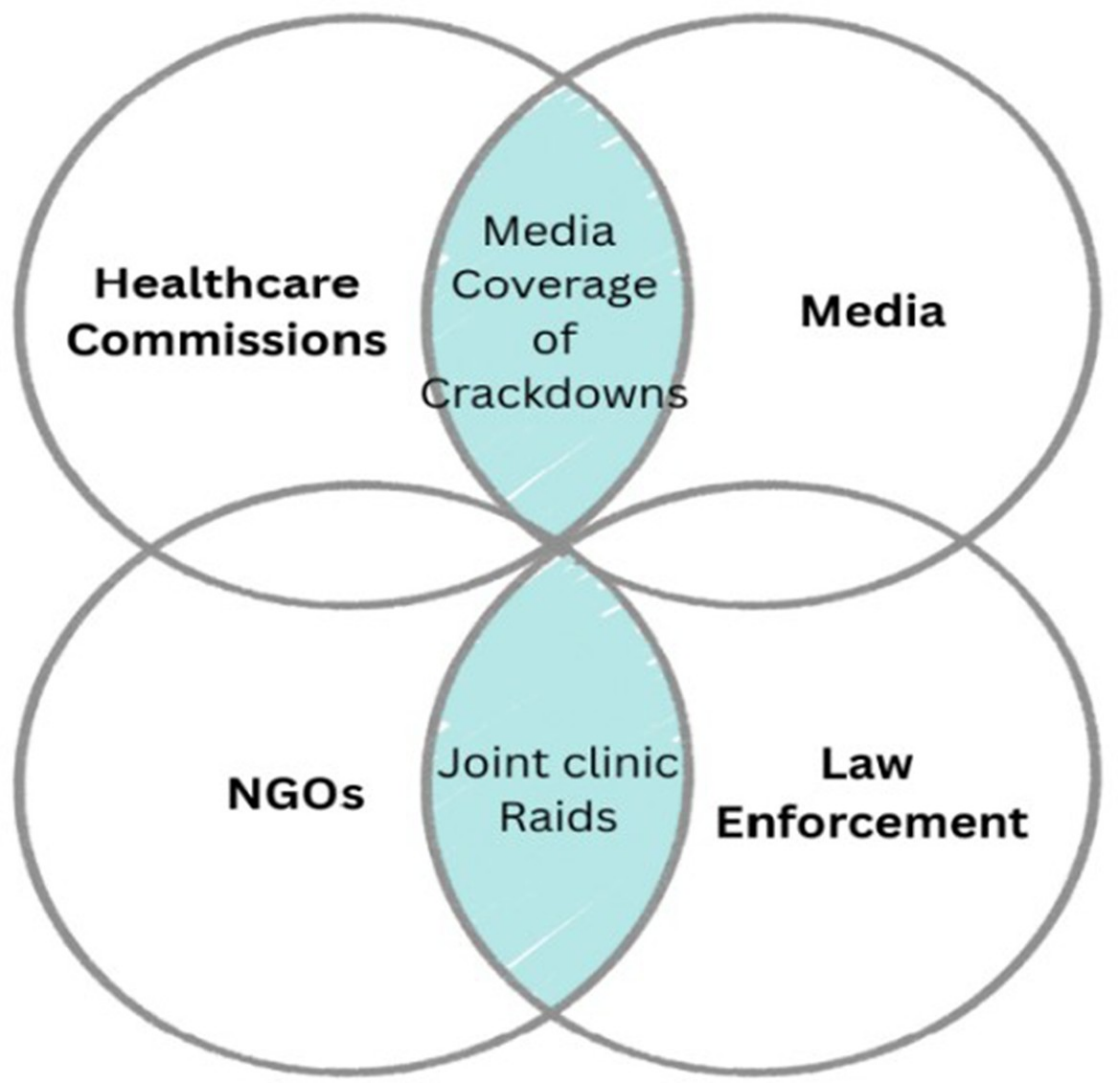
Joint awareness campaigns (e.g., IHRA effort)^[^48^]^.

By working with healthcare commissions and taking part in public awareness efforts, non-governmental organizations (NGOs) have supported anti-quackery campaigns, as shown in Figure 5^[^48^]^. Although there is less documentation of individual NGO-led initiatives, their participation in larger health education and community outreach programs helps raise public awareness of the risks associated with unlicensed medical professionals. For instance, in an effort to eradicate unlawful medical practices in the capital, the Islamabad Healthcare Regulatory Authority (IHRA) launched a thorough campaign against quackery with assistance from the District Health Office and local police^[^48^]^.

### Success so far

**Figure 6.**
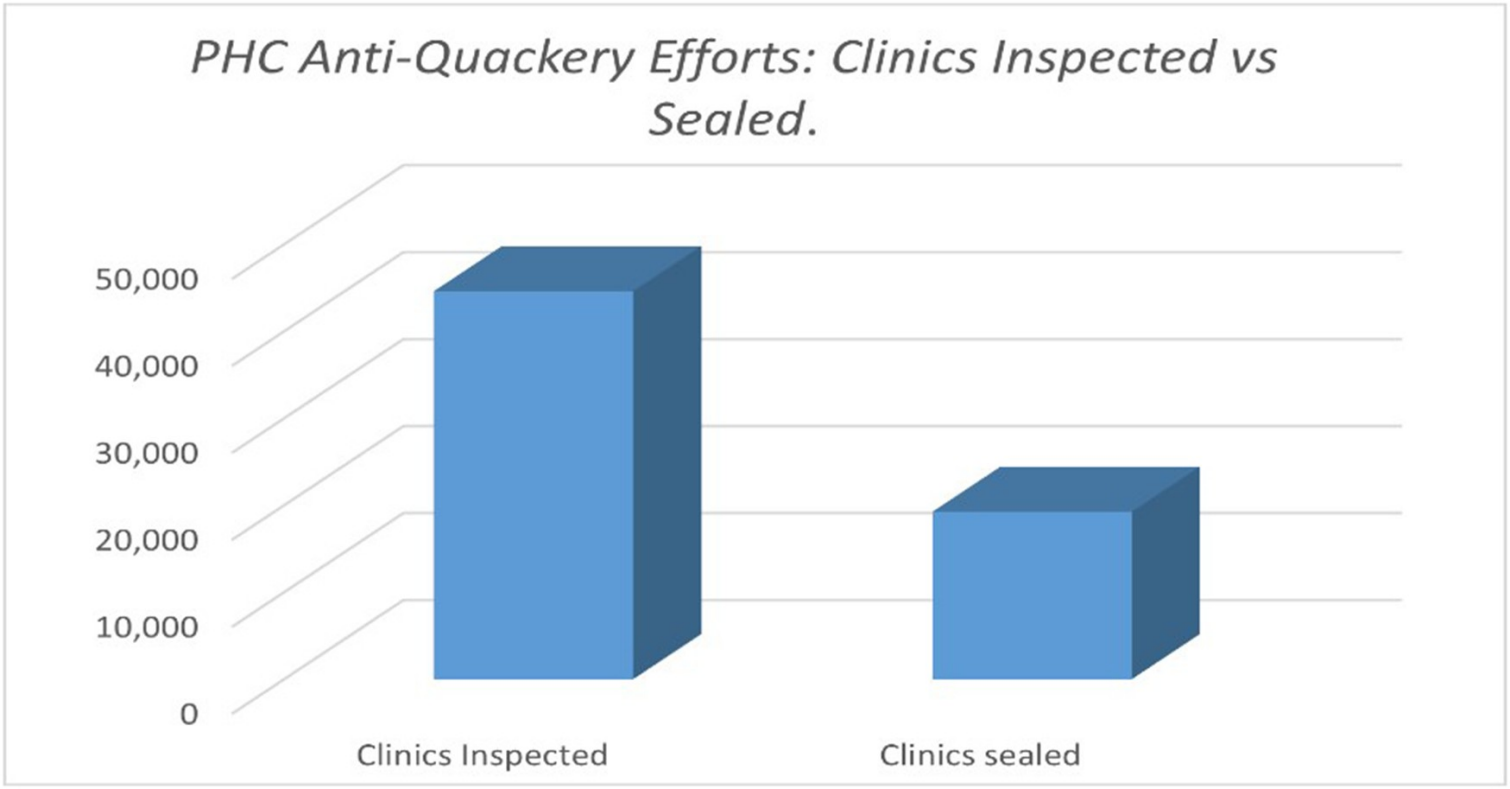
PHC anti-quackery efforts^[^8^]^.

The province’s healthcare quality has improved as a result of the PHC’s persistent efforts, which have closed thousands of unlicensed clinics and trained many medical personnel on minimal service delivery requirements (Fig. 6)^[^8^]^. Numerous quack clinics have been successfully identified and closed as a result of the SHCC’s focused operations in different districts, proving the efficacy of coordinated enforcement and public awareness campaigns^[^3^]^.

## Barriers to eliminating quackery

**Figure 7.**
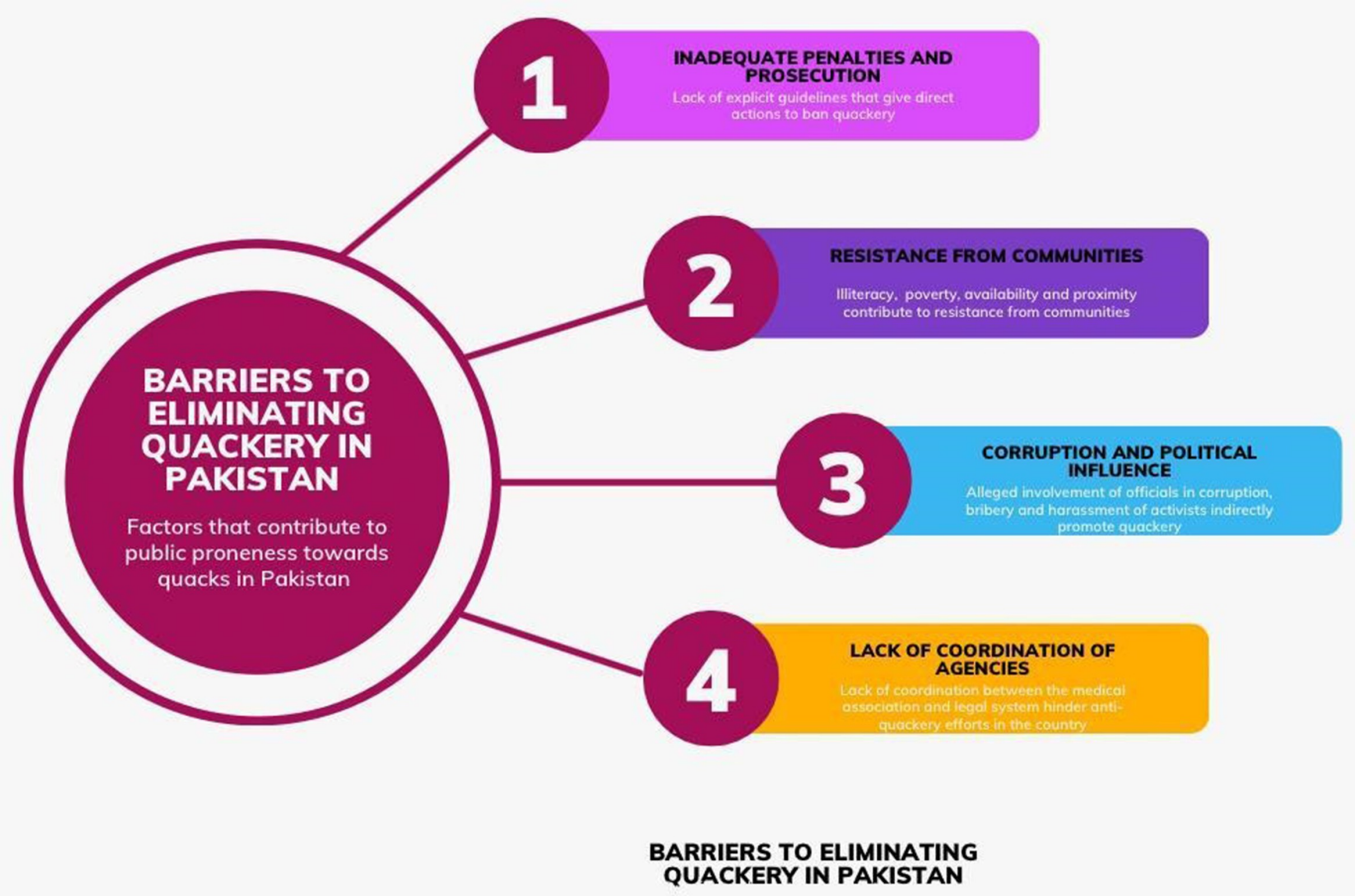
A conceptual illustration depicting barriers to eliminating quackery in Pakistan.

Pakistan has a long way to go in developing a healthcare system that is effective, accessible, and affordable^[^48^]^. In rural areas of Pakistan, quacks continue to dupe vulnerable and quackery-prone individuals for financial gain, which may lead to the loss of lives^[^49^]^. There are a number of reasons and factors that contribute to the public's proneness toward quacks, as conceptually summarized in Figure 7 and explained below.

### Inadequate penalties and prosecution

The healthcare commissions that were established in different provinces in Pakistan provide some regulatory standards for service delivery; however, enforcement of these regulatory standards has remained a challenge^[^43^]^. The current regulatory mechanisms have failed to implement a system to monitor and, hence, prevent unauthorized people from clinical practice^[^39^]^. Some District Health Offices (DHOs) initiated paperwork detailing the list of quacks in the districts, which was shared with the relevant public health department. However, no further action toward stopping the practices of quackery in these districts has been witnessed^[^43^]^.

According to the Punjab Healthcare Commission (PHC) anti-quackery strategy document, it has been a challenge identifying laws that address quackery in Pakistan in its exclusive terms, as no concrete data is available indicating the prevalence, manifestations, and actions taken to ban quackery^[^8^]^. A number of laws and regulations, however, directly or indirectly provide some mechanisms to take measures against quackery under the pretext of violation of certain clauses. These documents, however, lack the provision of any explicit guidelines that give direct actions to eliminate or ban quackery^[^8^]^. The success of regulatory bodies in curbing quackery is not up to the mark across the globe^[^30^]^.

### Resistance from communities

Health-seeking practices of communities in Pakistan are generally perceived as very poor by general practitioners (GPs)^[^42^]^. These practices can be attributed to the community’s illiteracy and extreme poverty, which make them vulnerable to seeking care from unlicensed medical practitioners (quacks)^[^43^]^.

A lot of patients are also impatient when seeking care. They end up visiting multiple providers over a short period of time, which poses a challenge and increases their chances of visiting quacks^[^43^]^. The long presence of quacks has led residents to trust them blindly because these quacks have developed strong bonds with residents over time^[^43^]^. Most patients in rural areas of Pakistan develop trust in quacks over time because these quacks tend to be accessible and available in emergency situations, as most hospitals and health centers are situated at a distance of about 30–35 km from many populated rural areas, and some only provide specialist care during morning hours^[^50^]^.

People prefer quackery formulations, which are products that, with no scientific backing, are promoted as effective treatments^[^49^]^. For several reasons, including cultural beliefs, cheaper rates, and over-the-counter availability, people prefer drugs that are prescribed by quacks^[^49^]^. The popular opinion of the public is that these powders are some kind of magical medication that alleviates patients’ symptoms in a very short period^[^49^]^.

### Corruption and political influence, along with a lack of coordination among agencies

In Pakistan, unqualified medical practitioners significantly outnumber licensed doctors, with estimates indicating the presence of one quack for every 350 individuals, while there is only one certified doctor available for every 1290 people (PMC)^[^11,51^]^. Due to limited healthcare infrastructure, many low-income families turn to quacks who provide easily accessible treatment at minimal charges, often allowing delayed payments or non-cash exchanges^[^8^]^.

Although health authorities have introduced a three-year jail sentence for practicing quackery, the law remains largely unimplemented. Accused individuals are frequently released by the courts, and the legal system struggles to hold supervising doctors accountable due to legal loopholes and ineffective enforcement^[^3^]^. Additionally, the Pakistan Medical Association (PMA) and media reports allege that some regulatory officials are involved in corruption, bribery, and harassment of activists, thereby indirectly promoting quackery^[^52^]^.

These systemic failures, combined with political interference and public dependency on informal healthcare, seriously hinder anti-quackery efforts in the country. As a result, institutional reform is urgently needed.

## Limitations and generalizability

This review relies on studies of variable quality and gray literature; therefore, quantitative prevalence estimates should be interpreted cautiously. This review has several limitations. First, it relies heavily on published and gray literature available in English, which may introduce publication and language bias and limit the inclusion of locally disseminated or vernacular data. Second, given the scarcity of nationally representative studies on quackery in Pakistan, many of the included findings are derived from localized reports and case studies, thereby constraining generalizability to the wider population. Third, the review did not apply formal meta-analytic or quantitative synthesis methods, as heterogeneity in study design and data reporting precluded direct comparison. Furthermore, the inclusion of media-sourced reports introduces the potential for reporting and selection bias, as such sources may overrepresent sensational cases while underreporting routine or undocumented instances of quackery. Besides this, the reliance on provincially reported data may limit generalizability, given disparities in surveillance systems, regulatory enforcement, and healthcare access across different regions of Pakistan. The operational definition and measurement of “quackery” remain inherently challenging within a pluralistic healthcare system where informal providers, traditional healers, and unlicensed practitioners often coexist, leading to potential misclassification and heterogeneity in the interpretation of findings. These factors may collectively influence the comprehensiveness and interpretability of the evidence presented.

From an ethical standpoint, it is important to acknowledge that, while unqualified or unregulated practitioners pose serious risks to public health, the abrupt elimination of these informal providers may inadvertently restrict access to basic healthcare for remote or underserved populations. Therefore, any regulatory intervention should adopt a phased and inclusive approach, balancing patient safety with the gradual strengthening of formal primary healthcare systems. This perspective underscores the need for policies that combine enforcement with capacity building and community engagement to achieve sustainable reform.

## Future directions and recommendations

Strengthening healthcare regulations revolving around licensure to practice has been tabled at the level of the provincial governments in recent years. The Punjab Healthcare Commission proposed an anti-quackery strategy implementation plan in 2015, which has been divided into short-, medium-, and long-term strategies.

### Short-term strategies

The short-term strategies focused on awareness campaigns among the general public and informing all stakeholders, including the Secretary of the Health Department, regarding the gravity of the issue. Furthermore, a provincial task force, headed by the Chief Minister, would be in charge of this campaign. Compulsory registration of all healthcare establishments and the formation of an anti-quackery cell were suggested as well.

### Medium-term strategies

In the medium term, sustained institutional and policy-level actions are essential to curb quackery and strengthen the healthcare delivery system. Efforts should focus on enhancing regulatory enforcement through the establishment of district-level monitoring units, periodic inspections, and digital registration of healthcare providers to ensure accountability and transparency. Integrating informal practitioners into structured training or certification programs can help upgrade their skills and gradually transition them into the formal health system under supervision. Simultaneously, public awareness campaigns should be institutionalized through collaboration between the Ministry of Health, district health authorities, media outlets, and community-based organizations to educate the public about the risks of unqualified treatment. Strengthening referral networks, improving access to affordable primary care, and incentivizing qualified practitioners to serve in underserved areas will further reduce reliance on quacks. These coordinated medium-term measures can bridge the current regulatory and service delivery gaps while setting the stage for sustainable long-term reform.

### Long-term strategies

Long-term strategies suggested legislative and systemic reforms, which included filling vacant doctor positions, rationalizing healthcare budget allocation across primary, secondary, and tertiary services, and supporting general practitioners through the Punjab Health Foundation to address service gaps in urban and peri-urban areas^[^8^]^. Similar strategies have also been developed by Khyber Pakhtunkhwa’s Healthcare Commission Regulations in 2022 and the Anti-Quackery Directorate, a frontline cohort of the Sindh Healthcare Commission established under the SHCC Act in 2013^[^3,8^]^.

However, the implementation of these strategies remains a challenge, as findings have reported a lack of administrative will among officials in the Punjab Health Commission to bring about actual change, a lack of critical decision-making, and untrained staff, along with inappropriate human resources to take action. A respondent shared, “All my knowledge about dealing with quacks in my area was gained on the job. When I was a new recruit several years ago, no one taught me how to identify and take action against these menaces.” Reportedly, Deputy District Health Officers appointed with the task had no prior experience in dealing with quacks, resulting in mishandling and improper reporting of medicines found during the sealing of premises. There was also a deficiency in procedural guidelines issued to field officers, and the anti-quackery cell formed by the Punjab Health Commission lacked an appropriate legislative framework, thus resulting in the cancellation of the unit by the Lahore High Court^[^27^]^. These challenges must be fully addressed in order to move closer to eradicating the deep roots of quackery in Pakistani society.

Disparities and needs can be analyzed to appropriately cater to them, which can counter the demand for quackery, and this can be achieved through GIS mapping. Lack of preventive care adds to the burden of the Pakistani healthcare system; therefore, a national and provincial health policy that prioritizes this would be beneficial. Appropriate legislation and frameworks for anti-quackery strategies are highly needed, along with the integration of services across public, private, and philanthropic sectors^[^53^]^. Moreover, enhancing the capacity of regulatory bodies such as SHCC and fostering more stakeholder collaboration is required^[^13^]^. Strict regulations against quackery and health professional registration, along with the recognition of courses and degrees being implemented, such as those mentioned in the PMDC Act 2023, are also steps in the right direction^[^54^]^.

An important yet underexplored strategy to mitigate quackery in underserved regions of Pakistan is the expansion of digital health and telemedicine services. Telemedicine platforms can bridge geographical and workforce gaps by connecting patients in remote areas with licensed physicians, thereby reducing reliance on unqualified practitioners. Government-supported initiatives, such as the Sehat Sahulat Program, could be further leveraged to integrate teleconsultation services, ensuring affordability and accessibility. Additionally, mobile health (mHealth) solutions and regulated e-clinics can facilitate timely diagnosis, triage, and referral, particularly in primary care settings. However, for effective implementation, robust regulatory oversight, digital literacy promotion, and infrastructure development, especially in rural areas, are essential to ensure quality, patient safety, and public trust.

Recent advancements in artificial intelligence, particularly large language models such as ChatGPT, have introduced new dimensions to healthcare delivery and public health discourse. A recent bibliometric analysis, Current Concerns and Future Directions of Large Language Model ChatGPT in Medicine: A Machine-Learning-Driven Global-Scale Bibliometric Analysis, highlights both the transformative potential and critical limitations of such technologies in medicine, including concerns related to accuracy, ethical use, and misinformation^[^55^]^.

In the context of quackery in Pakistan, these tools may serve a dual role: while they can enhance public awareness, patient education, and clinical decision support in resource-limited settings, they may also inadvertently contribute to the spread of unverified or misleading medical information if used without appropriate oversight. Therefore, integrating AI-driven tools into healthcare systems must be accompanied by robust regulatory frameworks, validation mechanisms, and professional supervision to ensure their safe and effective utilization in combating quackery and improving healthcare access.

## Conclusion

Quackery continues to pose a serious and persistent public health threat in Pakistan, undermining the credibility of the healthcare system and endangering countless lives. This narrative review highlights how unlicensed practitioners exploit systemic weaknesses – such as poverty, illiteracy, lack of regulation, and limited access to quality care – to establish a parallel and dangerous healthcare economy. Despite numerous crackdowns and awareness campaigns, quackery thrives in both rural and urban settings, often under the guise of traditional or alternative medicine. The consequences are far-reaching, including delayed treatment, complications, drug resistance, and increased healthcare costs. Addressing this issue requires a multi-faceted approach: strict enforcement of existing healthcare regulations, community-level education, improved accessibility to qualified medical professionals, and collaboration among government, media, and health institutions. Without immediate and coordinated action, quackery will continue to undermine public health efforts, perpetuate health disparities, and erode trust in the medical profession.

## Data Availability

The data supporting the findings of this study are available in the article’s references section. No additional datasets were generated or analyzed during the current study.

## Guaranter

Waseem Sajjad.
